# Protecting Citizens, Enabling Prevention: A Framework for Responsible Dietary Supplement Use in Europe

**DOI:** 10.3390/nu18050729

**Published:** 2026-02-25

**Authors:** Arrigo F. G. Cicero, Claudio Borghi, Federica Fogacci

**Affiliations:** 1 Hypertension and Cardiovascular Risk Research Center, Medical and Surgical Sciences Department, Alma Mater Studiorum University of Bologna, 40100 Bologna, Italy; claudio.borghi@unibo.it (C.B.); federica.fogacci@studio.unibo.it (F.F.); 2 Cardiovascular Medicine Unit, IRCCS AOU BO, 40138 Bologna, Italy; 3 Department of Medical Pharmacology, Medical Faculty, Ataturk University, Erzurum 25240, Turkey; 4 Italian Society of Nutraceuticals (SINut), 40100 Bologna, Italy

We welcome the Europe Commission’s growing ambition to protect cardiovascular health across the life course [1], and we would like to offer a constructive perspective on a topic that is often discussed in overly polarised terms, specifically, the role of food supplements containing well-characterised bioactives as part of primordial prevention—that is, keeping risk-factor levels low before disease pathways become established.

A useful way to frame this is through Geoffrey Rose’s population principle: “a large number of people at a small risk may give rise to more cases of disease than a small number who are at a high risk” [2].

The practical implication is that Europe’s most powerful prevention gains will come not only from identifying the highest-risk citizens [3], but also from gently shifting whole distributions—blood pressure, low-density lipoprotein cholesterol (LDL-C), glycaemia, adiposity—towards healthier ranges. In that same spirit, Rose cautioned that “it makes little sense to expect individuals to behave differently from their peers,” and encouraged policy that makes healthier choices easier and more “normal” [2].

Properly selected nutraceutical interventions—standardised, safe, and supported by reproducible clinical evidence on intermediate biomarkers—can help operationalise this “shift the curve” philosophy while respecting individual autonomy.

Blood pressure is, in our view, one of the clearest demonstrations of why this matters for population prevention. The European Society of Hypertension (ESH) defines a large segment of adults with high-normal office blood pressure (BP) (office systolic BP 130–139 mmHg and/or diastolic BP 85–89 mmHg)—values that do not meet traditional hypertension thresholds, yet are common, prognostically relevant, and modifiable [4]. This BP range is common in the general population—and it becomes even more so if one adopts the European Society of Cardiology (ESC) 2024 definition of “elevated BP” (office systolic BP 120–139 mmHg and/or diastolic BP 70–89 mmHg) [5].

In precisely this space—where lifestyle remains foundational, pharmacotherapy is not routinely pursued, and adherence to behavioural change is challenging at scale—nutraceuticals with credible evidence can offer a pragmatic adjunct. An ESH position document explicitly frames high-normal blood pressure as a target for strategies that may “prevent hypertension and/or cardiovascular complications,” noting that nutraceutical options may be candidates in a setting where reliable evidence for routine drug therapy is lacking [6]. In fact, even small downward shifts in systolic BP can translate into meaningful population benefits. This is because the prospective studies collaboration meta-analysis of individual-participant data (≈1 million adults, 61 prospective cohorts) showed a strong, approximately log-linear association between usual systolic BP and vascular mortality. Scaling this log-linear relationship implies that a 2 mmHg lower usual SBP corresponds to an estimated ~7% lower IHD mortality and ~10% lower stroke mortality [7].

Importantly, this is not an argument for indiscriminate supplementation. It is an argument for selectivity, distinguishing products that are merely popular from those that are standardised and clinically testable with plausible mechanisms and reproducible effects. The same ESH document emphasises that certain foods and natural products can lower blood pressure without major side effects and discusses nutraceutical options that are relevant to prevention pathways [6].

By way of concrete examples, the evidence base is particularly consistent for dietary nitrate sources such as beetroot juice, which in meta-analytic data show a mean systolic blood pressure reduction of around 4 mmHg in relevant populations [8]. More recently, the ESC guidelines suggest supplementing the diet with potassium [5].

Other interventions with supportive (though variable) evidence include magnesium supplementation in appropriate contexts, and selected bioactives such as aged garlic preparations, hibiscus, cocoa flavanols, and melatonin (especially when circadian or nocturnal blood pressure patterns are relevant)—always with the caveat that these are complements to lifestyle and, where indicated, pharmacotherapy [8]. In population terms, even modest average reductions can translate into meaningful reductions in incident hypertension and downstream cardiovascular events—exactly the kind of “primordial prevention dividend” that Rose envisioned [2].

In lipid management, a similarly constructive—yet rigorous—message emerges from the recent ESC/EAS focused update. It concludes that evidence that dietary supplements reduce cardiovascular risk is not compelling and therefore does not support supplements or vitamins for ASCVD risk reduction unless they have documented safety and clinically meaningful LDL-C-lowering efficacy. Rather than dismissing all “supplements” indiscriminately, the implication is that Europe should discourage products lacking robust standardisation, safety, and efficacy, while recognising selected interventions with reproducible effects on intermediate biomarkers [9].

This is best understood as a trust-building filter: a framework that protects citizens from variability and exaggerated claims, while leaving room for interventions that meet quality and evidence thresholds. A good example is the recognition that phytosterols, at typical doses, can lower LDL-C by around 10%, while guidelines appropriately note that outcome trials demonstrating cardiovascular benefit are lacking [10]. In addition, the European Food Safety Authority (EFSA) has supported a health claim to support the use of phytosterol as a lipid-lowering agent which is potentially effective in contributing to cardiovascular risk reduction [11]. That stance is neither permissive nor ideological; it is proportionate, evidence-calibrated, and aligned with the public’s legitimate expectation that “health products” should be consistent, standardised, and safe.

The work of the International Lipid Expert Panel (ILEP) has repeatedly tried to model this balanced approach. In its position paper on lipid-lowering nutraceuticals, the ILEP describes how certain nutraceuticals may help patients reach lipid goals and improve early vascular markers, while also underlining that the evidence base is heterogeneous and that these products are not substitutes for proven lipid-lowering pharmacotherapies—especially in high-risk settings [12]. The same paper frames practical clinical scenarios where evidence-based nutraceutical strategies may be considered (for example, moderate hypercholesterolaemia not meeting thresholds for drug therapy, or as supportive strategies when treatment intensity is limited by tolerability), again emphasising the need for an evidence-based approach and quality assurance [12]. Some dietary supplements with well-characterised lipid-lowering effects and favourable safety profiles include soluble fibre, bergamot polyphenolic fraction, artichoke extracts, garlic extract, berberine, fenugreek and some probiotics [13].

Another context where an effective early prevention could have a major public-health impact is the management of prediabetes. Twelve cohort studies (*n* = 547,287) were included, with follow-up ranging from 5.6 to 14.1 years. Compared with participants in the lowest estimated glucose disposal rate category, those in the highest category had a significantly lower risk of cardiovascular disease (HR 0.58; 95% CI 0.53–0.63), stroke (HR 0.62; 95% CI 0.56–0.69), and coronary heart disease (HR 0.46; 95% CI 0.25–0.83) [14]. Data from the large US Diabetes Prevention Program Outcomes Study (DPPOS) and the Chinese DaQing Diabetes Prevention Outcomes Study (DaQingDPOS) suggest that prediabetes remission is associated with a durable legacy effect spanning decades, with an approximately 50% reduction in the risk of cardiovascular death or hospitalisation for heart failure across diverse populations. Framing remission as a therapeutic target might open a new avenue for cardiovascular prevention [15]. Again, several meta-analyses of randomised clinical trials show an effective and persistent glucose-lowering effect of a number of dietary supplements, such as soluble fibre, chromium, vitamin D, cinnamon, berberine, fenugreek, resveratrol and some probiotics [16]. A paradigmatic example is Vitamin D. A meta-analysis of 10 randomised placebo-controlled clinical trials (*n* = 4478) concluded that, among subjects with low baseline vitamin D plasma levels (25-hydroxyvitamin D from 12 to 28 ng/mL) and prediabetes at the baseline, regression to normo-glycaemia occurred in 416/2253 (18.5%) participants allocated to vitamin D supplementation versus 312/2225 (14.0%) allocated to placebo. Across trials, effect estimates favoured vitamin D (RR 1.09–12.6). The pooled RR for regression to normoglycemia was 1.27 (95% CI 1.12–1.45), with minimal heterogeneity (*I*^2^ = 1%) [17].

We would also encourage Europe to view nutraceuticals through a systems lens, recognising that cardiovascular prevention increasingly overlaps with metabolic health and liver health. Non-alcoholic fatty liver disease is common and tightly interwoven with cardiometabolic risk; notably, cardiovascular disease is a major driver of adverse outcomes in populations with metabolic dysfunction-associated steatotic liver disease (MASLD). An ILEP position paper on MASLD discusses how nutraceuticals may have roles as adjuncts to diet and lifestyle in selected circumstances, while carefully noting evidentiary limitations and the need for robust evaluation [18]. A policy narrative that supports only well-studied, standardised, safe bioactives—rather than “anything sold as a supplement”—would therefore not only align with cardiovascular prevention but also reinforce coherent messaging across related chronic disease agendas.

In heart failure—where the stakes are high and therapeutic substitution would be strongly inappropriate—an ILEP position paper stresses that nutraceuticals should be considered only as additions to optimal guideline-directed therapy, and only where the evidence supports safety and potential benefit [19]. This is precisely the kind of disciplined, citizen-protective posture that European policy should amplify. Conversely, citizens (and the institutions) should also be aware that supplementation of high-dose of ubiquinol appears safe for heart failure patients and, when given on top of standard of care, has been associated with improvement of heart functionality, reduction in the risk of hospitalisation because of heart failure and reduction in risk of mortality; therefore, it is an evidence-based and cost-effective supplementation [20].

For all these reasons, we respectfully propose that the European Commission’s prevention narrative explicitly recognises the potential role of rigorously studied food supplements—those containing standardised bioactives with demonstrated safety and reproducible effects on intermediate risk markers—particularly in areas where population-level shifts are feasible and impactful, with blood pressure in the general population as the clearest example [21]. This recognition need not dilute scientific standards; on the contrary, it can strengthen them by setting expectations for quality, standardisation, transparency, and appropriate use, while discouraging products that do not meet these criteria.

## Data Availability

Not applicable.
